# Referees

**DOI:** 10.1002/rmb2.12362

**Published:** 2021-01-18

The publication of invaluable papers in the Reproductive Medicine and Biology depends on the prompt, careful review of submitted manuscripts. We would like to thank the Editorial Board members, the Editorial Advisory Board members and the following experts for reviewing manuscripts from November 1, 2019 to October 31, 2020.

1

Ashok Agarwal

Hidenori Akutsu

Yasuhisa Araki

Yoshiyuki Asai

Atsushi Azumaguchi

Tsuyoshi Baba

Aldo E. Calogero

Koji Chiba

Akihisa Fujimoto

Masakatsu Fujinoki

Toshihiro Fujiwara

Maki Fukami

Aisaku Fukuda

Shinichiro Fukuhara

Atsushi Fukui

Tatsuro Furui

Gustavo Gonzales

Maki Goto

Miyuki Harada

Shu Hashimoto

Yuji Hirao

Kenichiro Hiraoka

Yasushi Hirota

Masashi Iijima

Masahito Ikawa

Shuntaro Ikeda

Osamu Ishihara

Tomomoto Ishikawa

Nobuhiko Itami

Junya Ito

Shoichiro Iwatsuki

Shikha Jain

Takeshi Kajihara

Haruhiko Kanasaki

Satoru Kanto

Yukiko Katagiri

Fuminori Kimura

Kazumi Kishida

Satoshi Kishigami

Michio Kitajima

Hideyuki Kobayashi

Akira Komiya

Shinnosuke Komiya

Koji Kugu

Hiroki Kurahashi

Keiji Kuroda

Hirohisa Kyogoku

Ryo Maekawa

Hiromichi Matsumoto

Yasuhiro Matsuoka

Keiko Mekaru

Hiroyuki Minoura

Hidehiko Miyake

Toshinobu Miyamoto

Satoshi Mizuno

Tetsunori Mukaida

Noriko Nakamura

Akitoshi Nakashima

Hiroki Nakata

Mitsuru Nishiyama

Mariko Ogawa

Hajime Oishi

Hidetaka Okada

Konosuke Okada

Masanori Ono

Makoto Orisaka

Satoko Osuka

Kuniaki Ota

Hideaki Sawai

Koichiro Shimoya

Masahide Shiotani

Hiromitsu Shirasawa

Toshihiro Tajima

Seido Takae

Toshifumi Takahashi

Satoru Takamizawa

Toshiyuki Takeshita

Kazuhiro Takeuchi

Isao Tamura

Fuminori Taniguchi

Hisanori Taniguchi

Hidetaka Tasaki

Hiroshi Uchida

Takashi Umehara

Akihiro Umezawa

Hitoshi Ushijima

Teruhiko Wakayama

Yasuhisa Yamashita

Kaoru Yanagida

Atsumi Yoshida

Hiroaki Yoshida

Ai Yoshihara

Osamu Yoshino

